# Association of youth climate worry with current and past mental health symptoms: A longitudinal population-based study

**DOI:** 10.1192/j.eurpsy.2023.636

**Published:** 2023-07-19

**Authors:** F. Vergunst, C. Prentice, M. Orri, H. Berry, F. Vitaro, R. Treblay, S. Cote, M. C. Geoffroy

**Affiliations:** ^1^University of Oslo, Oslo, Norway; ^2^University of Oxford, Oxford, United Kingdom; ^3^McGill University, Montreal, Canada; ^4^Macquarie University, Sydney, Australia; ^5^University of Montreal, Montreal, Canada; ^6^Pediatrics, University of Montreal, Montreal, Canada

## Abstract

**Introduction:**

Young people are worried about climate change but the association between climate worry and current and past mental health has not been examined in population-based samples.

**Objectives:**

To examine 1) the prevalence of worry about climate change at age 23-years and its association with contemporaneous mental health symptoms, and 2) and adolescent mental health symptoms.

**Methods:**

We used a Canadian population-based birth cohort (n=1325) to examine associations between 1) climate change at age 23-years and concurrent anxiety, depression, and suicidal behaviors, and 2) mental health at age 15 and 17 years defined as anxiety, depression, aggression-opposition, inattention-hyperactivity. We adjusted for participants’ socioeconomic status, childhood IQ, sex, and parental history of psychopathology.

**Results:**

Most participants were worried about climate change: 190 (14.3%) were extremely worried, 553 (41.7%) were somewhat worried, 383 (28.9%) were very worried, and 199 (15.0%) were not at all worried. Worry about climate change was associated with significantly elevated contemporaneous anxiety, depression, and suicidal thoughts. In longitudinal analysis, adolescent anxiety was associated with higher climate change worry at age 23-years while adolescent aggression-opposition was associated with lower climate change worry.

**Conclusions:**

Worry about climate change is associated with contemporaneous mental health symptoms. However, longitudinal analysis suggests that this is largely explained by prior mental health, with adolescent anxiety symptoms linked with higher worry and aggression-opposition with lower worry. Future studies should aim to better define the dimensions of climate anxiety and track it alongside symptoms using prospective follow-up studies.

**Disclosure of Interest:**

None Declared

